# Supplementary material for: A high-resolution mRNA expression time course of embryonic development in zebrafish
Source: eLife. 2017 Nov 16;6:e30860. doi: 10.7554/eLife.30860 (PMC5690287; doi:10.7554/eLife.30860)
Supplement: Supplementary file 6. [file elife-30860-supp6.zip › biolayout-clusters-files/Cluster147-genes.html]

Cluster147


# Cluster147: Genes

| Ensembl ID | Gene Name | Chr | Start | End | Biotype |
| --- | --- | --- | --- | --- | --- |
| ENSDARG00000078853 | arhgef19 | 23 | 24211336 | 24240819 | protein\_coding |
| ENSDARG00000062978 | ccdc151 | 6 | 8014843 | 8029212 | protein\_coding |
| ENSDARG00000001825 | cfap43 | 13 | 24587430 | 24612701 | protein\_coding |
| ENSDARG00000010270 | fezf1 | 25 | 27829706 | 27832889 | protein\_coding |
| ENSDARG00000031337 | hoxa10b | 16 | 21104367 | 21107184 | protein\_coding |
| ENSDARG00000007009 | hoxa11b | 16 | 21098935 | 21101559 | protein\_coding |
| ENSDARG00000023031 | hoxa2b | 16 | 21120854 | 21123364 | protein\_coding |
| ENSDARG00000030110 | myod1 | 25 | 30843777 | 30846017 | protein\_coding |
| ENSDARG00000014050 | ngfb | 6 | 49170767 | 49217174 | protein\_coding |
